# A Hidden Risk of Levodopa: A Case Report of Drug-Induced Syndrome of Inappropriate Antidiuretic Hormone Secretion (SIADH) and Hyponatraemia

**DOI:** 10.7759/cureus.95587

**Published:** 2025-10-28

**Authors:** Salwa Haddy, Pankaj Singh, Elodia Brunetti, Juan Pedro Simon-Turriate

**Affiliations:** 1 Medicine, East Surrey Hospital, Redhill, GBR; 2 Geriatrics, East Surrey Hospital, Surrey and Sussex NHS Trust, Redhill, GBR

**Keywords:** care of the elderly, hyponatraemia, levodopa-carbidopa, oral levodopa, parkinson' s disease, syndrome of inappropriate secretion of antidiuretic hormone (siadh)

## Abstract

Hyponatraemia is a common electrolyte imbalance in hospitalised patients, particularly among the elderly. While often mild, asymptomatic, and largely self-resolving, severe cases can lead to significant neurological complications. We report a 67-year-old woman with Parkinson’s disease and chronic hyponatremia who developed acute symptomatic hyponatraemia after starting levodopa/carbidopa. Her sodium levels dropped to 108 mmol/L, with associated confusion. Investigations revealed euvolemic hyponatraemia consistent with SIADH, with normal adrenal and thyroid function. Levodopa/carbidopa was suspected as the trigger and discontinued. She was transitioned to rotigotine, resulting in gradual correction of sodium levels and full neurological recovery. This case highlights a rare but important adverse effect of levodopa, emphasising the need for clinicians to consider dopaminergic medications as a potential cause of hyponatraemia. Early recognition and appropriate management are crucial to prevent serious complications, particularly in older adults with pre-existing electrolyte disturbances.

## Introduction

Hyponatraemia, defined as a serum sodium level below 135 mmol/L, is one of the most frequently encountered electrolyte imbalances in clinical settings [1]. It varies in presentation and severity, and while many individuals remain asymptomatic, others may develop symptoms such as headache, nausea, vomiting, lethargy, or changes in mental status. In more serious cases, complications can become life-threatening [2]. Though the causes of hyponatraemia are broad, medications are a well-recognised contributor, particularly in elderly patients who often face the challenge of multiple coexisting medical conditions and polypharmacy [3]. Levodopa, a cornerstone in the management of Parkinson’s disease, has been infrequently linked to hyponatraemia, with only a few cases documented in the medical literature [4]. Here, we describe the clinical course of a 67-year-old woman who developed acute symptomatic hyponatraemia soon after increasing levodopa/carbidopa therapy. Within one week of increasing the medication, the patient sustained a fall resulting in a femoral fracture requiring hospital admission. Her hospital stay was complicated by acute hyponatraemia, accompanied by confusion and poor response to standard treatments. Careful reassessment of her recent medication history and a re-evaluation of her Parkinson’s diagnosis led to the discontinuation of levodopa/carbidopa. Subsequently, her sodium levels returned to baseline, accompanied by full neurological recovery. This case is shared with the intention of increasing awareness around the potential for levodopa-induced hyponatraemia and highlighting how iatrogenic complications, particularly in older adults, can be avoided through timely recognition and intervention.

## Case presentation

A 67-year-old woman was admitted to the hospital following a mechanical fall that resulted in a left neck of femur fracture. The fall involved tripping over an object, falling onto her left side, and striking her head against a cupboard. There was no loss of consciousness, vomiting, chest pain, or preceding symptoms. On examination, she was found to have an irregular heart rhythm, and her left leg appeared shortened and externally rotated, consistent with a fracture. Imaging confirmed a displaced intracapsular fracture of the left femoral neck. Her pre-operative cognitive screen was intact (4AMTS 10/10). She was independently mobile prior to admission, living with her husband, and received no social care support. 

She had a background of Parkinson’s disease, atrial fibrillation, hypothyroidism, and long-standing chronic hyponatraemia. Her regular medications included apixaban, levothyroxine, bisoprolol, and co-beneldopa (Madopar). The Madopar was prescribed as one controlled-release co-beneldopa 100 mg/25 mg nightly, in addition to a four-times-daily dose of 50/200mg of co-beneldopa throughout the day (0600, 1000, 1400 and 1800 hours). This had been increased to five times daily to improve her evening tremors that were affecting her with the aim to shorten her 'off-period', a week prior to her fall. The on-off period in Parkinson’s disease refers to the fluctuation between times when medication is working ('on' period), where symptoms and movements (e.g., stiffness, slowness, tremors) are well controlled, and times when the effect wears off (‘off’ period). Over time, as the disease progresses, there are often shorter 'on' periods and may require adjustment of medications to help with symptom management [5]. 

The patient underwent a left hip cemented hemiarthroplasty on 29/08/2024. Post-operatively, she was commenced on low molecular weight heparin for venous thromboembolism prophylaxis, with plans to transition back to her long-term anticoagulant, apixaban, for atrial fibrillation. However, by 01/09/24, she developed acute confusion with an acute decrease of serum sodium, from 120 mmol/L to 108 mmol/L, on a background of chronic hyponatraemia (baseline 120-125 mmol/L). A CT head showed no intracranial haemorrhage and no other causes for the acute confusion. She was transferred to the High Dependency Unit (HDU) for close monitoring. Despite chronic hyponatraemia being part of her medical background, this marked decline in sodium levels was new. 

On physical examination, her fluid status appeared euvolemic; there was no indication of dehydration or fluid overload. Laboratory results showed a serum osmolality of 221 mmol/kg, urine osmolality of 278 mmol/kg, urine sodium at 23 mmol/L, and urine potassium at 28.7 mmol/L. Morning serum cortisol levels ranged from 363 to 652 nmol/L, and thyroid function tests remained within the normal range. These findings helped rule out adrenal insufficiency and thyroid dysfunction as causes of her hyponatraemia. Altogether, the clinical and biochemical picture was consistent with euvolemic hyponatraemia, most likely secondary to the Syndrome of Inappropriate Antidiuretic Hormone Secretion (SIADH). After a thorough review of the literature and a detailed assessment of her medication history, co-beneldopa was identified as a likely contributor to the acute drop in sodium levels. Her other medications were also reviewed and ruled out as contributing, as she had been on these long-term, and they were not associated with hyponatraemia. Although uncommon, there is evidence that levodopa may stimulate antidiuretic hormone (ADH) release, resulting in SIADH-like effects. Given this possibility and her acute confusion with refusal for oral medications, her usual Parkinson’s regimen, co-beneldopa, was temporarily substituted with a once daily transdermal rotigotine (dopamine agonist) patch on 01/09/24, as an alternative treatment for Parkinson’s symptoms (Table 1). 

**Table 1:** Laboratory results on admission

Test	Patient results	Normal range
Serum sodium (mmol/L)	120	133-146
Serum potassium (mmol/L)	4.3	3.5-5.3
Serum osmolality (mmol/kg)	246	275-295
Urine osmolality (mmol/kg)	278	50-1200
Thyroid-stimulating hormone (µIU/L	1.24	0.35-4.94
Free thyroxine (pmol/L)	11.8	9.0-19.1
Serum cortisol (nmol/L)	520	138-635

In HDU, she was closely monitored in terms of clinical observation, neurological signs and bloods and her sodium levels improved, returning to her baseline of 120 mmol/L within a couple of days (on 03/09/24), and her mental state normalised (4AMTS 10/10). 

She was stepped down from HDU to the orthogeriatrics ward, where she continued to be monitored with daily bloods and clinically, during which time she remained stable. She was noted to have slightly decreased calcium levels (2.17 mmol/L) and borderline Vitamin D levels (51 nmol/L) and started on Adcal D3. After two further days of monitoring, she was assessed by the occupational and physical therapists, including stairs assessments, which she passed and was deemed medically fit for discharge. She was discharged back to her own home with safety netting, mobilisation advice and plans for review of electrolyte bloods in the community and an outpatient Dual Energy X-ray Absorptiometry (DEXA) scan. Her sodium levels, reviewed three weeks later, were 132 mmol/L, and she was entirely asymptomatic. She subsequently had a further outpatient appointment with the geriatricians for a clinical review of the patient as well as her Parkinson’s disease medication and her DEXA results, with no further concerns. 

This case serves as a reminder that dopaminergic medications such as levodopa may rarely cause hyponatremia and should be considered in the differential diagnosis, especially in patients with pre-existing electrolyte imbalances and complex medication regimens. In such situations, alternatives like rotigotine may offer a safer therapeutic option.

## Discussion

Hyponatraemia is defined as a serum sodium concentration below 135 mmol/L and is one of the most frequent electrolyte disorders in both acute and chronic care settings [1]. Among hospitalised patients, the prevalence is estimated to be between 15% and 30%, with even higher rates observed in older adults [6]. This increased risk in elderly patients is often attributed to multimorbidity and the use of multiple medications [7]. The condition can be categorised as mild, moderate, or severe. Severe hyponatraemia carries the risk of significant complications, including delirium, seizures, coma, and even death [2]. 

In SIADH, certain drugs cause the body to release too much ADH or make the kidneys overly sensitive to it. This leads to excess water reabsorption in the kidneys, which dilutes the sodium in the blood, resulting in hyponatraemia (low blood sodium). Levodopa-induced hyponatraemia is believed to result from inappropriate secretion of ADH [8]. Experimental studies have demonstrated that dopaminergic stimulation may enhance ADH release from magnocellular neurons [9]. However, this system appears to be highly adaptable, which might explain why only some individuals develop this rare side effect. This variability underscores the importance of individual patient factors and idiosyncratic responses. 

In reviewing existing literature, we found only a few previous case reports linking levodopa/carbidopa to hyponatraemia. One involved a 68-year-old man who developed the condition after initiating therapy with levodopa, carbidopa, and amantadine [10]. Another described a 73-year-old woman who developed symptomatic hyponatraemia four days after starting levodopa [11]. More recently, a case report highlighted a dose-dependent relationship between levodopa and hyponatraemia in a patient who presented with hiccups. Interestingly, there is limited literature presenting or reporting levodopa-induced hyponatraemia in individuals not diagnosed with Parkinson’s disease [12], suggesting that dopaminergic pathways alone may be sufficient to trigger the response in certain susceptible individuals.

## Conclusions

Severe hyponatraemia is an uncommon but potentially life-threatening side effect of levodopa/carbidopa therapy. This case study illustrates how important it is to remain vigilant about medication-induced complications, especially in elderly patients, where the risk of harm is heightened by existing comorbidities, polypharmacy and long-term electrolyte imbalances. Early recognition of the association between levodopa and hyponatraemia allowed for timely intervention in this case, with complete resolution of symptoms following medication adjustment. Clinicians should consider alternatives like rotigotine in managing Parkinson’s disease when hyponatraemia becomes a concern. Ultimately, proactive medication review and individualised care can play a critical role in preventing serious iatrogenic events.
